# Supplementary material for: Spatiotemporal interplay between multisensory excitation and recruited inhibition in the lamprey optic tectum
Source: eLife. 2016 Sep 16;5:e16472. doi: 10.7554/eLife.16472 (PMC5026466; doi:10.7554/eLife.16472)
Supplement: Source code 1. — DOI: http://dx.doi.org/10.7554/eLife.16472.017 [file elife-16472-code1.docx]

**Matlab code**

**For visualization of electrophysiological traces:**

clc

close all

clear

a = input('enter file number for gapfree >> ' , 's');

b = strcat(a,'.abf');

[gapfree si h] = abfload(b); % define file

x = gapfree(:,:);

% current-clamp

[nx ny] = size(x);

recording = x(:,1:3:ny);

% recording = recording(:,1:2:end);

stimulus1 = x(:,2:3:end);

stimulus2 = x(:,3:3:end);

[nx ny] = size(recording);

si = 50*10^-6;

total_time = length(gapfree)*si;

tout = 0:si:total_time;

for i = 1:ny

subplot(311)

plot(tout(1:end-1),recording(:,i))

subplot(312)

plot(tout(1:end-1),stimulus1(:,i))

subplot(313)

plot(tout(1:end-1),stimulus2(:,i))

disp(i)

pause

end

inclusion = [1:ny];

mean_trace = mean(recording(:,inclusion)');

mean_trace_subsample = mean_trace(1:20:end);

recording_subsample = recording(1:20:end,:);

tout_subsample = tout(1:20:end-1);

figure,plot(tout_subsample(1:end),recording_subsample(:,inclusion),'k','LineWidth',1)

hold on

plot(tout_subsample(1:end),mean_trace_subsample,'r','LineWidth',2)

xlim([0 3])

ylim([-80 0])

hold on

**compute total conductance**

clear

close all

clc

R_in = 2..*10^9;

IV_b = -63.3516*10^-3 -0.01;

tau = 0.12;

load tout

tout = tout(1:1:end-1)';

aa = find(tout > 1.165 & 1.1652 < tout);

tstart = aa(1);

aa = find(tout > 1.254 & 1.255 < tout);

tend = aa(1);

load 052

z = mean(recording(:,:)');

clear recording

Vm1 = z(tstart:tend)'*10^-3- 0.01;

Vm1 = smooth(Vm1,20);

load 053

z = mean(recording(:,:)');

clear recording

Vm2 = z(tstart:tend)'*10^-3- 0.01;

Vm2 = smooth(Vm2,20);

tout = tout(tstart:tend);

I_inj1 = mean(Vm1(1:500)/R_in);

I_inj2 = mean(Vm2(1:500)/R_in);

k = 0;

dVmdt1 = diff(Vm1)./diff(tout);;

dVmdt2 = diff(Vm2)./diff(tout);

Cm = tau/R_in;

for i = 2:length(Vm1)

k = k + 1;

b1 = I_inj1 - Cm*dVmdt1(k);

b2 = I_inj2 - Cm*dVmdt2(k);

B = [b1 b2]';

%%%%%%%%% direct extraction of reversal potential and total conductances

% G_total(i) = (b1+b2)/(Vm1(i)+Vm2(i));

% V_rev1(i) = Vm1(i) - (1./G_total(i))*b1;

% V_rev2(i) = Vm2(i) - (1./G_total(i))*b2;

V_rev(i) = (b2*Vm1(i) - b1*Vm2(i))/(b2-b1);

G_total(i) = b1/(Vm1(i)-V_rev(i));

%%%%%%%%%%

a11 = Vm1(i) + 0.075;

a12 = Vm1(i) - (-.00);

a21 = Vm2(i) + 0.075;

a22 = Vm2(i) - (-.00);

A = [a11 a12; a21 a22];

x = mldivide(A,B);

Gi(i) = x(1);

Ge(i) = x(2);

end

lower_x = find(tout > 0.15 & tout < 0.151);

upper_x = find(tout > 0.279 & tout < 0.28);

figure,plot(tout,smooth(Gi,10),'b',tout,smooth(Ge,10),'r')%,tout,G_total,'k')

**For spiking properties and input resistance:**

clear all

close all

clc

a = input('enter file number for IV >> ' , 's');

b = strcat(a,'.abf');

[x] = abfload(b); % define file

x = x(:,:);

[nx ny] = size(x);

y = x(:,1:2:ny);

stimulus = x(:,2:2:ny);

[nx ny] = size(y);

clear x

x = y;

si = 20000;

sampling_frequency = 20000; % 20KHz

sampling_interval = 1/sampling_frequency; % step

L = length(x); % size of time series

tout = (0:1:L-1)*sampling_interval;

% spike_count = zeros(ny,1);

for i = 1:ny

disp(i)

[y_no50hz(:,i) spike_count(i) spike_time{i}] = spike_occurence(x(:,i));

end

%tout(spike_time{i})

set(0,'DefaultAxesColorOrder',[1 0 0;0 1 0;0 0 1; 0 0 0;],...

'DefaultAxesLineStyleOrder','-')

% visual inspection of spike detector performance

for i = 1:ny

plot(tout,x(:,i)),

hold on

d = length(spike_count(i));, spike_amp = 20*ones(1,d)*i;

if d > 0, plot(spike_time{i},spike_amp,'k.'), end

disp(i)

pause

% close

end

%%%%%%%%%%%%%% CURRENT VERSUS SPIKE FREQUENCY AND MAX FREQ

%%%%%%%%%%%%%% ADAPT VALUES BELOW

figure,plot(tout,x)

initial = input('enter intial deflection >> ');

pause

step = input('enter step >> ');

current_injections = initial + step*(0:ny-1);

initial_spike = input('enter intial spiking of interest >> ');

figure,plot(current_injections(initial_spike:end),spike_count(initial_spike:end),'.')

xlabel('current injection')

ylabel('spike count')

last_spike = input('enter last spiking of interest >> ');

figure,plot(current_injections(initial_spike:last_spike),spike_count(initial_spike:last_spike),'.')

xlabel('current injection')

ylabel('spike count')

%%%%% INPUT RESISTANCE %%%%%%%%

pause

IV_low_end = input('enter trace number at low end for R computation > ');

IV_high_end = input('enter trace number at high end for R computation > ');

R_traces = x(:,IV_low_end:IV_high_end);

[nxx nyy] = size(R_traces);

for i = 1:nyy

baseline(i) = mean(R_traces((0.001:0.1)*20000,i));

end

disp('Mean of baseline')

disp(mean(baseline))

disp('')

disp('Std of baseline')

disp(std(baseline))

disp('')

figure, plot(tout,R_traces)

time_start = 0.13;

time_end = .49;

R = x(time_start*si:time_end*si,IV_low_end:IV_high_end); % changed from x

T = tout(time_start*si:time_end*si);

figure,plot(T,R)

[nx ny] = size(R);

figure

for i = 1:ny

[fit, yy] = physfit('expc', T,R(:,i));

yy1 = cell2mat(yy);

hold on

pause

plot(T, yy1,'r', T, R(:,i),'k')

res = fit.p;

time_constant(i) = -1/res(2);

voltage_for_IV_curve(i) = fit.p(1)*exp(fit.p(2)*1) + fit.p(3);

end

figure,plot(current_injections(IV_low_end:IV_high_end),voltage_for_IV_curve,'.')

xlabel('current pA'), ylabel('voltage mV')

I_inj = current_injections(IV_low_end:IV_high_end);

V_mem = voltage_for_IV_curve;

brob = robustfit(I_inj,V_mem);

y = brob(1)+brob(2)*I_inj;

R_in = brob(2);

R_b = brob(1);

hold on

plot(I_inj,brob(1)+brob(2)*I_inj,'k')

brob_g = robustfit(V_mem,I_inj);

g_b = brob_g(1);

g_slope = brob_g(2);

disp('Input resistance')

disp(R_in)

disp('IV intercept')

disp(R_b)

disp('rest conductance')

disp(g_slope)

disp('g_intercept')

disp(g_b)
